# Problem Gambling in the Fitness World—A General Population Web Survey

**DOI:** 10.3390/ijerph17041342

**Published:** 2020-02-19

**Authors:** Anders Håkansson, Artin Entezarjou, Göran Kenttä, Fernando Fernández-Aranda, Susana Jiménez-Murcia, Björn Gunnarsson

**Affiliations:** 1 Faculty of Medicine, Dept of Clinical Health Sciences Malmö, Primary Care, Lund University, S-205 02 Malmö, Sweden; artin.entezarjou@med.lu.se (A.E.); bfmgunnarsson@gmail.com (B.G.); 2 The Swedish School of Sport and Health Sciences, S-114 86 Stockholm, Sweden; goran.kentta@gih.se; 3 School of Human Kinetics, University of Ottawa, Ottawa, ON K1N 6N5, Canada; 4 Swedish Sport Federation, 100 61 Stockholm, Sweden; 5 Pathological Gambling Unit, Dept Psychiatry, Bellvitge University Hospital-IDIBELL, 08907 Barcelona, Spain; ffernandez@bellvitgehospital.cat (F.F.-A.); sjimenez@bellvitgehospital.cat (S.J.-M.); 6 Ciber Fisiopatología Obesidad y Nutrición (CIBERObn), Instituto de Salud Carlos III, 28029 Barcelona, Spain; 7 Dept Clin Sciences, Faculty of Medicine, University of Barcelona, 08007 Barcelona, Spain

**Keywords:** gambling, sports psychology, behavioral addiction

## Abstract

The world of sports has a complex association to problem gambling, and the sparse research examining problem gambling in athletes has suggested an increased prevalence and particularly high male predominance. The present study aimed to study frequency and correlates of problem gambling in populations with moderate to high involvement in fitness or physical exercise. This is a self-selective online survey focusing on addictive behaviors in physical exercise distributed by ‘fitness influencers’ on social media and other online fitness forums to their followers. Respondents were included if they reported exercise at least thrice weekly, were above 15 years of age, and provided informed consent (N = 3088). Problem gambling, measured with the Lie/Bet, was studied in association with demographic data, substance use, and mental health variables. The occurrence of lifetime problem gambling was 8 percent (12 percent in men, one percent in women). In logistic regression, problem gambling was associated with male gender, younger age, risky alcohol drinking, obsessive-compulsive disorder, and less frequent exercise habits. In conclusion, in this self-recruited population with moderate to high fitness involvement, problem gambling was moderately elevated. As shown previously in elite athletes, the male predominance was larger than in the general population. The findings strengthen the link between problem gambling and the world of sports.

## 1. Introduction

Problem gambling is a global condition affecting mental health, with great variability in prevalence between 0.1 and 5.8 percent across measures and settings [1]. Importantly, this range includes the clinical definition of gambling disorder on a diagnostic level, which has a prevalence of approximately 0.5 percent [2]. Problem gambling has been shown to be approximately three to four times more common in men than in women [3,4].

Being an athlete, particularly on an elite level, has been suggested to be a risk factor of problem gambling, although limited research can support this notion. Grall-Bronnec and coworkers reported a problem gambling frequency of eight percent in male athletes [5]. Moreover, a recent study including both male and female elite athletes revealed an altogether moderately elevated frequency of lifetime problem gambling, but with a striking gender difference (14 percent in men, compared to 1 percent in women). Male gender is known to be a risk factor of problem gambling; however, this association appeared to be even more pronounced in this study of elite athletes [6].

Traditional sport participation and high-level exercise in various domains may potentially be associated with specific personality traits, such as a sensation-seeking and competitive behavior [7,8], and a potential association between high engagement in physical training and addictive behaviors has been discussed in this sense. In student athletes, although in one study problem gambling was not more common than in other students [9], other research has associated this population with a number of other health hazards [10]. Also, it has been shown that problem gambling in this group of highly sports-involved young individuals may be associated with higher expectations of gambling outcome and less of enjoyment-related attitudes towards gambling [11].

Further, the potential link between sports and gambling may be further enhanced by the large involvement of the gambling industry and intense advertising in the world of sports [12]. In addition to this, high-profile athletes have been used in marketing, and an increasing body of literature in recent years has highlighted the potential association between sports and problem gambling [13]. Recent years have seen an increasing popularity in sports betting, particularly in the younger population [14], and possibly perceived as a socially acceptable form of gambling [15,16]. Also, sports betting is common among treatment-seeking problem gamblers [17], and this therefore further contributes to a perceived link between those who are attracted to sports and gambling. This development may further lead to the hypothesis that sports involvement and problem gambling may be associated.

The culture of elite sports may influence values, norms and training behaviors in other populations engaged in fitness and exercise, since high-performance athletes often become role models for good and bad. It has been suggested that three distinct relationships may exist between exercise and psychiatric disorders: (1) athletes may be highly successful in spite of a coexistent primary psychiatric disorder, (2) athletes may have chosen training and exercise as means of coping with the disorder, and (3) athletes may have psychiatric illness triggered by training itself [18]. Moreover, the existence of exercise addiction in populations highly engaged in fitness training [19] may indicate an increased occurrence of other addictive behavior in this population. Based on the findings from the sparse literature on problem gambling in elite athletes, with a higher prevalence and particularly high male predominance, it may be of importance to study problem gambling in broader groups of individuals involved in sports or with extensive fitness habits. Altogether, the associations between various training levels, various performance levels and problem gambling may be complex; while some data indicate an increased problem gambling prevalence in athletes, as described above, problem gambling in the general population has been associated with unhealthy behaviors, including a more sedentary behavior [20]. These contrasting findings suggest a complex association between exercise level and problem gambling that needs more attention. For these reasons, the present self-selective web-based general population survey aimed to study the frequency of problem gambling and its correlates in individuals who exercise at least thrice weekly, and who are recruited through web forums with a pronounced emphasis on exercise and fitness.

## 2. Methods

The present study was conducted using an open web questionnaire distributed through a number of social media forums focusing on fitness, and intuitively focused mainly on individuals with a large focus on fitness and sports, although without intentionally addressing elite athletes specifically. In the present study, the questionnaire was sent to key individuals behind these web forums (i.e., high-profile fitness personalities), who distributed the survey in their information channels, mainly to individuals following their communication in social media. The survey was introduced as an open self-test for potential exercise dependence (data to be reported in a separate publication) and its correlates. Based on the present findings of problem gambling in athletes, the present analysis rather focuses on variables associated with problem gambling in this population.

Following the link distributed online, the survey was described as related to potentially addictive exercise behavior and addressed individuals above 15 years of age who exercise at least thrice weekly. Thus, whenever an individual entered the link to the survey, she/he was provided with a written information about the study, and the questionnaire opened only if the individual provided consent to the study. The survey was openly available on the internet and collected no data which could identify specific individuals (such that age was reported in age categories and no geographical information was collected). No financial compensation was given to the respondents.

Problem gambling was assessed as occurring at any time during lifetime, and measured through the two-item Lie/Bet scale, which includes one question about increased gambling (measuring the diagnostic criterion describing tolerance) and one question about having to lie to one’s nearest family about the extent of one’s gambling (corresponding to the diagnostic criterion describing lying), and where the endorsing of at least one item is interpreted as a positive screen for problem gambling [21,22,23]. The same instrument was also used in the study of problem gambling prevalence in European athletes cited above [5], allowing for comparison with the prevalence data of that study. The Lie/Bet instrument has been described to have a high sensitivity (0.81) and specificity (0.94) with respect to problem gambling as measured with the well-established Problem Gambling Severity Index in clinical mental health samples [24].

In order to identify potential correlates of problem gambling, a number of other variables were assessed in the questionnaire. These included a self-report of the individual’s lifetime history of being diagnosed with a number of common disorders (‘have you ever been diagnosed with….’?), with respect to depression, eating disorders, generalized anxiety disorder, social phobia, panic disorder, obsessive-compulsive disorder, or AD/HD (attention-deficit/hyperactivity disorder) or ADD (attention-deficit disorder), as well as a question about whether the individual had ever felt the need to seek treatment for psychological distress. In addition, questions were asked about whether the respondent had used each or the following substances during the past year; cannabis, benzodiazepines, cocaine, amphetamine, opioids, stimulant medications, or anabolic androgenic steroids. Also, hazardous alcohol drinking was screened for (with a past-year time frame) using the abbreviated AUDIT screen (Alcohol Use Disorder Identification Test), including the three consumption-related questions (the AUDIT-C). These questions include frequency of alcohol drinking, number of standard drinks on a typical day, and frequency of binge drinking [25]. The Cronbach alpha for the AUDIT-C has been reported to be 0.75 in the present setting, with a high test-retest reliability of 0.93 [26]. Toner and co-workers recently summarized studies assessing the AUDIT-C (and other instruments related to alcohol consumption) in young people, with an average sensitivity of 0.83, an average specificity of 0.70, and an average reliability of 0.92, and described this instrument as being at least promising as a brief screening tool for alcohol problems [27]. Other questions included gender, age, daily smoking and the number of days of exercise per week (3–7 days) for a typical week. (Table 1).

A total of 3413 responses confirmed informed consent and age above 15 years. Among these, 101 subjects reported exercise on less than three days per week and were excluded and did not continue the survey. Among the remaining 3281 subjects, gambling variables were available for 3088 subjects, who were finally included in the analyses. Among included respondents, 64 percent were men (n=1971) and 36 percent (*n* = 1117) were women. Two percent (*n* = 49) were in the youngest age group (15–18 years), 13 percent (*n* = 403) were 19–24 years, 22 percent (*n* = 670) were 25–29 years, 33 percent (*n* = 1009) were 30–39 years, 23 percent (*n* = 697) were 40–49 years, seven percent (*n* = 226) were 50–59 years, and one percent (*n* = 34) were 60 years or older.

The study was approved by the Regional Ethics Board, Lund, Sweden (file number 2017/822).

### Statistical Methods

All analyses were carried out in SPSS version 22 (IBM SPSS Statistics for Windows, Armonk, NY: IBM Corp.). The first analyses compared the problem gambling group (with at least one item endorsed on the Lie/Bet screening tool) to all remaining respondents, with respect to a number of socio-demographic variables and variables describing health variables and exercise. These univariate analyses were carried out with chi-square test for categorical variables, and the Mann-Whitney test for one continuous variable (the AUDIT-C score for alcohol consumption) which was judged not to be equally distributed. In a second analysis, all variables demonstrating a significant association with problem gambling in univariate analyses were entered simultaneously in a logistic regression analysis, using problem gambling as the dependent variables. Variables with multiple options (age groups, number of days of exercise, and the AUDIT-C scores) were included as continuous variables, i.e., calculated odds ratios refer to the associations seen with increasing age groups, increasing number of days of exercise and increasing AUDIT-C score). For all measures, 95 percent confidence intervals were used, i.e., the level of significance was set at *p* < 0.05.

## 3. Results

In total, 7.8 percent (*n* = 241) were problem gamblers, endorsing either the lying criterion (4.4 percent, *n* = 137), or the tolerance criterion (5.8 percent, *n* = 179). The reporting of problem gambling was markedly more common among males than among females (12 vs. 1 percent, chi-square = 117.64, *p* < 0.0000001). The frequency of problem gambling was the highest among subjects who reported three or seven days of exercise (11 and 10 percent, respectively), and lower in groups reporting four, five or six weekly days of exercise (nine, six and six percent, respectively). In binary comparison of problem gamblers and the remaining respondents, problem gambling was significantly associated with male gender, age group, cannabis, cocaine and amphetamine use, alcohol consumption, daily smoking, and having been diagnosed with an eating disorder, an obsessive-compulsive disorder, or AD/HD/ADD (Table 1).

The logistic regression included male gender, age groups, number of days of exercise, cannabis use, cocaine use, amphetamine use, eating disorder, obsessive-compulsive disorder, ADHD/ADD, smoking, and the AUDIT-C score, controlling these variables for one another. Here, problem gambling remained positively and significantly associated with male gender, alcohol consumption, and obsessive-compulsive disorder, and negatively and significantly associated with age and days of exercise (Table 2).

## 4. Discussion

The present study, in a sample of self-selected survey respondents recruited by means of online fitness profiles, demonstrated a moderately elevated lifetime frequency of problem gambling, and a high level of male predominance in problem gamblers.

The 8 percent occurrence is comparable to the figure reported in European team sports athletes (8 percent), measured with the same screening tool as in the present study but in almost exclusively male respondents [5], and elite athletes in various sports in Sweden (7 percent, measured instead with a three-item screening tool, the NODS-CLiP) [6,28]. The marked gender difference was also consistent with previous data in elite athletes; the present study did not demonstrate a likely increase in female problem gambling (one percent) compared to the general population, whereas the figure in men was 12 percent, comparable to the prevalence figure reported from elite athletes (14 percent, [6]) but clearly higher than that reported in Grall-Bronnec’s study of team elite athletes, where almost 100 percent of participants were men [5].

In the present study, somewhat unexpectedly, no association was seen with a lifetime history of being diagnosed with depression. This finding is in contrast to the association described in previous literature, and which has been seen in cross-sectional data [29], and a high prevalence of co-morbid affective disorders, including depression, is common in clinical samples of patients seeking treatment for a gambling disorder [30]. Another variable which was unrelated to problem gambling—in the final regression model—was eating disorders, although it was inversely associated with problem gambling in the non-adjusted model. Eating disorders, which are more prevalent in women, have received attention in attention to fitness involvement in women [31]. In contrast, as problem gambling in the present study was closely associated with male gender, it can be assumed that the negative association with problem gambling disappeared due to the adjustment for gender in the model.

In contrast, both risky alcohol drinking and a diagnosed obsessive-compulsive disorder were associated with problem gambling. For both alcohol use and obsessive-compulsive disorder, a previous association with problem gambling has been seen [29]. For alcohol drinking, this association must be seen as well-established in different populations, at least in males [32,33], and therefore this association may not be surprising in the present study. In contrast, in the previous study on elite athletes in the present setting, problem gambling surprisingly was not associated with the level of risky alcohol consumption [6]. While this association may be specific to individuals in high-level sports involvement, it was not seen in this population, likely taking a position between elite athletes and the general population. Thus, like in many other populations, alcohol drinking in adults with high degree of physical exercise should be seen as a likely correlate of problematic gambling behavior.

At the same time, the link between problem gambling and obsessive-compulsive disorder in the present study should be interpreted with some caution, as the item is endorsed by a small proportion of subjects in the study. However, although seen as separate diagnostic constructs, authors have suggested that addictive behaviors and obsessive-compulsive disorder may share some characteristics [34], and both conceptual and genetic similarities have been documented [35]. Thus, it cannot be excluded that individuals with a problematic gambling behavior have been diagnosed with an obsessive-compulsive condition which either is a condition on its own, co-occurring with the gambling problem, or which has been difficult to outline as a separate condition in individuals with a compulsive gambling behavior. In addition, previous data from treatment-seeking patients have demonstrated that the personality trait persistence may be overrepresented in patients seeking help for sports betting [16]; thus, personality traits pointing in the same direction as the obsessive-compulsive symptomatology could potentially be related to the world of sports studied here, and merits further research in studies where the type of gambling also can be controlled for.

The predominance of men in the group of problem gamblers is far from surprising; one consistent finding is that a clear majority of problem gamblers are males, both in clinical settings and in the general population. However, the male-to-female prevalence ratio for problem gambling or gambling disorder is typically in the range of 3:1 or 4:1 [3,4], including in clinical samples in the present geographical setting [17,36]. In contrast, here, 12 percent of men and only one percent of women in the present sample were problem gamblers, similar to a previous study in elite athletes where 14 and one percent of men and women, respectively, were classified as problem gamblers [6]. Thus, like in elite athletes, the risk of problem gambling in individuals with a high degree of involvement in physical exercise may not be elevated in women, but markedly elevated in men. One previous finding is that pathological gambling behavior in women develops later in life, although more rapidly with a shorter time from start of gambling to onset of problem gambling [37,38,39], and that women with a manifest gambling disorder are therefore older than their male counterparts [17,40].

However, the present study does not entirely address younger adults, and the large gap in frequency between genders is somewhat surprising but also similar to that of previous research in elite athletes [6]. More research may be needed in order to elucidate the reasons for the particular predominance of male gambling-related problem behavior in these physically active populations, and whether the link between sports behavior and gambling behavior is particularly pronounced in men. For example, while problematic sports betting appears to be more common in males than in females [17,41], it remains to be understood whether this contributes to the marked gender difference in problem gambling within the world of sports and fitness.

Although no association remained when controlling for all other significant variables, specific drugs of abuse were significantly more common in problem gamblers than in other respondents. For comparison, although specifically in the young, studies in student athletes have demonstrated the same overall picture, with an association between problem gambling and comorbid substance use and health risk behaviors [10]. In the present study, the association with problem gambling was seen for cocaine, amphetamine and cannabis, whereas no association was seen with opioids, benzodiazepines, or anabolic androgenic steroids. This indicates that problem gambling in a population of physically active individuals may be more closely linked to certain types of substance use than to others. The stimulating types of drugs (although not stimulant medications) previously have been linked to problem gambling; cocaine has been linked to problem gambling in the community [42] as well as in the criminal justice system [43], and the misuse of prescription stimulants also has been associated to gambling disorder and to other impulse-related conditions [44]. Likewise, some evidence of an association between problem gambling and cannabis has been described previously, although the research in the area is limited [45]. Also, despite relatively clear differences between problem gamblers and the remaining subjects in the present study, the associations with each of the drugs studied here disappeared in the adjusted analysis. The same is true for smoking, which was somewhat more commonly reported by problem gamblers in the present study, although this association did not persist in the adjusted analysis. Daily smoking was relatively uncommon in the present population of individuals involved in fitness and can be seen as a representation of an unhealthy behavior in contrast to the exercise in this population. Tobacco smoking is known to be one of the unhealthy behaviors statistically associated with problem gambling in the general population [20] but did not clearly demonstrate to be an independent risk factor in the present analyses.

The present study may have implications for preventive and screening policies in the world of sports and in the population. Like in elite athletes, males were markedly more likely to be problem gamblers. This may call for specific preventive efforts in men with an involvement in regular fitness and sports. While these groups may seem to have a health-oriented lifestyle, an increasing attention has been paid to psychological distress and mental disease in sports athletes [46], which likely has implications also for individuals who are on a sub-athlete level but still with a high degree of involvement in physical exercise, as can be suspected from the population studied here. Also, and specific to the gambling field, the sports involvement itself may make individuals more prone to extensive gambling than for the general population, particularly given the popularity of sports betting in recent years [15] and the high prevalence of sports betting in individuals seeking treatment for problem gambling [17]. Also, based on research from student athletes, it is likely that attitudes and expectancies related to gambling may separate problem gamblers from others [11], i.e., risk factors which could potentially be addressed through educational or motivational efforts. Researchers have called for increased attention to problem gambling in the world of elite athletes [13], and the present web survey in people likely to have a high-degree involvement in physical exercise calls for an increased attention to male problem gambling also in this group.

## 5. Limitations

The present study has limitations, mainly linked to the fact that inclusion in the study is based on self-selection to an entirely open survey in an online setting, with study responses originating from individuals choosing to enter an online link in order to take the test. While the present paper focuses on problem gambling, the link to the present study primarily attracted individuals with a relatively high involvement in fitness and sports, and signalled a focus on addiction-like exercise patterns and health issues related to this. Thus, a completely open web survey as this one is likely to attract an overrepresentation of individuals with a particular interest in this area, such that findings are difficult to generalize to the general population. For example, many respondents received the link to the survey through social media. Higher use of social media has been shown to be associated with a higher likelihood of mental health problems [47], indicating that people who were reached by the present study survey may display an overrepresentation of psychological distress. Also, the present study addressed gambling with a screening tool for problem gambling but did not include specific data about the type of gambling involvement, including whether it was primarily land based or online gambling. Future studies should focus more specifically on detailed gambling patterns in relation to sports and fitness involvement. In addition, this two-item screening tool, the Lie/Bet, has limitations compared to a more extensive screening or diagnostic questionnaire. Also, its time frame, assessing a lifetime history of the variables included, has limitations compared to longer instruments providing a past-year diagnosis. However, the use of the Lie/Bet had the purpose of providing a screen appropriate for the rapid web survey format, and it allows for the comparisons with the prior study in European athletes [5], where the same brief instrument was used.

## 6. Conclusions

In conclusion, in individuals with a likely high involvement in sports and fitness, as previously described in elite athletes, an elevated occurrence of problem gambling is seen. As shown previously in elite athletes, gender differences were particularly pronounced, with the risk increase for problem gambling virtually seen only in males. As expected from the general population, problem gambling was linked to alcohol drinking, younger age, as well as obsessive-compulsive disorder. Certain drugs of abuse were more common in the problem gambling group, although statistical associations disappeared in the final model. These findings call for further research and preventive measures with respect to problem gambling in the world of sports and fitness.

## Figures and Tables

**Table 1:** Comparison of problem gamblers and non-problem gamblers. Demographic characteristics, substance use and psychological distress.

	Problem Gamblers (>0 Lie/Bet Items, *n* = 241), % (*n*)	Non-Problem Gamblers (0 Lie/Bet Items, *n* = 2847), % (*n*)	*p* Value
Male Gender	94 (227)	61 (1744)	<0.001
Age Group			<0.001
- 15–18 yrs	1 (3)	2 (46)	
- 19–24 yrs	18 (44)	13 (359)	
- 25–29 yrs	30 (72)	21 (598)	
- 30–39 yrs	36 (87)	32 (922)	
- 40–49 yrs	13 (32)	23 (665)	
- 50–59 yrs	1 (2)	8 (224)	
- 60+ yrs	0 (1)	1 (33)	
Days of Exercise/Week			
- 3	17 (42)	12 (338)	<0.01
- 4	29 (71)	27 (757)	
- 5	27 (65)	33 (940)	
- 6	16 (38)	21 (592)	
- 7	10 (25)	8 (220)	
Cannabis	15 (36)	7 (195)	<0.001
Benzodiazepines	2 (6)	2 (43)	0.24
Cocaine	6 (15)	2 (64)	<0.001
Amphetamine	4 (9)	1 (39)	<0.01
Opioids	7 (17)	6 (174)	0.56
Stimulants Medications	1 (3)	1 (32)	0.87
Anabolic Androgenic Steroids	2 (5)	1 (33)	0.22
Sought Treatment for Psychological Distress	29 (69)	30 (846)	0.72
Eating Disorder	1 (3)	4 (115)	0.03
Panic Disorder	8 (18)	6 (171)	0.36
Social Phobia	2 (6)	2 (69)	0.95
Generalized Anxiety Disorder	8 (19)	6 (174)	0.28
Obsessive-Compulsive Disorder	2 (6)	1 (22)	<0.01
AD/HD or ADD	5 (11)	2 (51)	<0.01
Depression	15 (37)	15 (441)	0.96
Daily Smoking	7 (18)	4 (128)	0.04
AUDIT-C, Median (Interquartile Range) *	4 (3–6)	3 (2–5)	<0.01

* upper confidence interval below 1.00 (rounded off to two decimals).

**Table 2:** Variables associated with problem gambling (defined as the endorsing of at least one Lie/Bet item). Logistic regression.

	OR	95% Confidence Interval	*p* Value
Male Gender	9.25	5.17–16.57	<0.001
Older Age Group	0.79	0.70–0.88	<0.001
Number of Days of Exercise Weekly	0.88	0.78–1.00 *	0.04
Cannabis	1.16	0.72–1.74	0.63
Cocaine	1.21	0.56-2.63	0.63
Amphetamine	1.20	0.46–3.15	0.71
AUDIT-C Score	1.16	1.09–1.24	<0.001
Daily Smoking	1.27	0.72–2.23	0.41
Eating Disorder	1.64	0.47–5.70	0.44
Obsessive-Compulsive Disorder	3.32	1.18–9.35	0.02
ADHD/ADD	2.03	0.98–4.20	0.06

* upper confidence interval below 1.00 (rounded off to two decimals).
